# Nourishment in Typhoid, Fever

**Published:** 1860-09

**Authors:** 


### NOURISHMENT IN TYPHOID FEVER.

English journals and English books contain much strong language and many strong facts in favor of nourishment in typhoid fever. A belief in the necessity of sustaining the strength of those who are to pass through this dangerous disease, is certainly gaining ground. But there are still some who cannot see that the debility which follows the fever is even worse than the fever itself, or, more properly speaking, are so intent upon the disease, that they forget the condition produced by it. Upon the prostration depend tardy convalescence and many of the sequelæ which not only add greatly to the patient's sufferings, but threaten life itself.

It is clear that many patients relish certain kinds of food, and bear them well, but are kept upon disagreeable and comparatively innutricious articles of diet, the use of which is sanctioned by old and unfounded theories. As the French have advocated every variety of treatment, and been quite as heroic as any physicians, it gives us pleasure to furnish a summary of some remarks by Dr. Monneret, physician of Neckar Hospital, in Paris, published in the *Bulletin de Therapeutique* for January 30th, 1860. After denying that there is any special treatment for the disease, he strongly insists upon the necessity of sustaining the strength by liquid and solid alimentary substances. He commences with an emetic, which is repeated on the second day, if the action previously has not been sufficient. During the second, third, and fourth days he gives lemonade, bark, quinine, wine and broth, and soon after strong soups. He remarks that the physician may feel some repugnance at giving wine and soup to a patient who has a dirty tongue, diarrhœa, fever and delirium, but on reflection it will be seen that there is no other contra indication to the employment of alimentary substances. Those who insist upon a strict diet are still influenced by the doctrine of irritation. They see inflammation where it does not exist, and are constantly afraid of exciting or increasing it. They therefore allow patients to die, who might recover if properly nourished. Whatever may be said about fever, it should not prevent us from sustaining the strength. Do we not prolong the existence of phthisical persons, undermined by fever, by nourishing them until the last moment? Do we not, by nourishment, sustain, for a long time, the life of persons laboring under visceral diseases?

Surgeons have learned, although rather late, and at the expense of their patients, that fasting is often pernicious after great operations. A great number of complications of all kinds assail the sufferers and compromise their existence, if the strength is not upheld by broths, wine, and even more substantial aliments.

Besides, all fears, inspired by systematic ideas founded in bad medical doctrines, fall before impartial observation, which show us that patients who are a prey to fever, digest very well all the things mentioned. Still more, diarrhœa, gurgling, and meteorism, far from augmenting, partially diminish.

We can easily conceive that alimentation should be without harm, as the stomach and a great part of the small intestine are exempt from all textural if not functional change.

Patients attacked by a form of fever which progresses rapidly and violently, terminating fatally, perhaps, in less than a week, take wine and broth with great pleasure, and bear them better, perhaps, than medicated drinks.

In following the proposed course, we only act in accordance with the universally admitted law that nature is to be assisted. In this disease a want of strength is what we have great reason to fear, and, certainly, nothing can so effectually guard a patient against this danger as the use of nutrient materials, which are easy of digestion.

M. Piorry writes in the same strain, in the *Gazette des Hopitaux* for March, 1860. He gives the following rules:

Give, in general, nourishment, when patients wish and need it.

Choose that which observation has shown to be the most suitable and most easily digested.

Commence with a small quantity.

Observe the effects, and increase the quantity promptly, if the experiments show such a course to be desirable.—*Boston M. and S. Journal.*

